# Novel Piezoelectric Paper‐Based Flexible Nanogenerators Composed of BaTiO_3_ Nanoparticles and Bacterial Cellulose

**DOI:** 10.1002/advs.201500257

**Published:** 2015-10-27

**Authors:** Guangjie Zhang, Qingliang Liao, Zheng Zhang, Qijie Liang, Yingli Zhao, Xin Zheng, Yue Zhang

**Affiliations:** ^1^ Department of Materials Physics and ChemistryUniversity of Science and Technology BeijingBeijing100083P.R. China; ^2^ Key Lab for New Energy and NanotechnologyUniversity of Science and Technology BeijingBeijing100083P.R. China

**Keywords:** bacterial cellulose, barium titanate, energy harvester, nanogenerators, piezoelectric paper

## Abstract

A piezoelectric paper based on BaTiO_3_ (BTO) nanoparticles and bacterial cellulose (BC) with excellent output properties for application of nanogenerators (NGs) is reported. A facile and scalable vacuum filtration method is used to fabricate the piezoelectric paper. The BTO/BC piezoelectric paper based NG shows outstanding output performance with open‐circuit voltage of 14 V and short‐circuit current density of 190 nA cm^−2^. The maximum power density generated by this unique BTO/BC structure is more than ten times higher than BTO/polydimethylsiloxane structure. In bending conditions, the NG device can generate output voltage of 1.5 V, which is capable of driving a liquid crystal display screen. The improved performance can be ascribed to homogeneous distribution of piezoelectric BTO nanoparticles in the BC matrix as well as the enhanced stress on piezoelectric nanoparticles implemented by the unique percolated networks of BC nanofibers. The flexible BTO/BC piezoelectric paper based NG is lightweight, eco‐friendly, and cost‐effective, which holds great promises for achieving wearable or implantable energy harvesters and self‐powered electronics.

## 1 Introduction

With the increasing demand for sustainable and reliable energy for personal electronics and wireless nanosystems, it is envisioned as a promising approach to harvest energy from the ambient sources, such as body movements, air flow, acoustic waves, and even thermal fluctuations which are available in most of the circumstances.^1^, ^2^, ^3^, ^4^ Nanogenerators (NGs) are emerging as novel devices which can convert various kinds of ambient energy into electric power via piezoelectric,^5^ triboelectric,^6^, ^7^ or pyroelectric effect^4^ both in nanoscale and macroscale. As a typical piezoelectric nanomaterial, ZnO nanowire was found to demonstrate piezoelectric potential distributing along its *c*‐axis when subjected to lateral bending or axial tension and compression, which is the basic mechanism of piezoelectric NGs.^8^, ^9^ Up till now, ZnO nanowires based piezoelectric NGs not only have been used for harvesting mechanical energy with much enhanced output performance^10^ but also functioned as self‐powered sensors to detect motion and vibration signals.^11^, ^12^

In the pursuit of higher piezoelectric output performance, it is desirable to utilize piezoelectric materials with higher piezoelectric coefficient. Conventional piezoelectric ceramics including Pb(Zr_1−*x*_Ti*_x_*)O_3_ (PZT), BaTiO_3_ (BTO), and (1−*x*)Pb(Mg_1/3_Nb_2/3_)O_3−*x*_PbTiO_3_ (PMN‐PT) has been outstanding candidates for high‐output NGs in view of their excellent piezoelectric properties.^13^, ^14^, ^15^, ^16^, ^17^ In particular, BTO is the most attractive material for its lead‐free property as well as high piezoelectricity.^16^ However, as a major problem for practical application, these bulk piezoelectric ceramics are intrinsically very brittle and thus hardly compatible with the irregular mechanical deformations. To address this problem, it has emerged as an effective methodology to incorporate piezoelectric nanomaterial into soft polymer matrix resulting in a flexible piezoelectric nanocomposite to improve the mechanical durability of the piezoelectric components.^18^ Polydimethylsiloxane (PDMS) is the most commonly used polymer matrix because of its flexibility and robustness. Piezoelectric polymer such as poly(vinylidene fluoride) (PVDF)^19^ and its copolymers^20^ have also been utilized as matrix because they themselves are piezoelectric materials which can provide enhancement for the overall output performance. For the design of piezoelectric composite film, it is believed that piezoelectric nanomaterial must be dispersed uniformly in the composite to obtain optimized piezoelectric output.^21^ However, it is challenging to get such a well‐distribution because most of the flexible matrix materials used are often sticky and hydrophobic, which makes it hard for ceramic nanoparticles to separate sufficiently. In view of these difficulties, adding dispersants such as graphitic carbons^18^, ^22^ or metal nanorod^23^ into the composite and biosynthesis of piezoelectric nanostructure by virus templates^24^ to realize entangled network structures have shown promising enhancement. Despite the enormous performance improvement that has been achieved, more facile, inexpensive, and scalable approaches are still needed.

As a kind of natural material, cellulose has become an attractive candidate for paper based devices due to its flexibility, biocompatibility, and low cost. Recently, various techniques have been explored to implement novel paper based devices. Compared to other flexible materials, cellulose possess a much lower coefficient of thermal expansion, which is an advantage for the thermal stability of devices.^25^ In addition, suitability for printed electronic device is another promising merit for paper materials.^26^ Bacterial cellulose (BC), a kind of biopolymer produced by Gluconacetobacter strains, has higher mechanical strength and better chemical stability than regular paper due to its high purity and crystallinity. Due to its intrinsic textured nanofibrillated structure, BC can present itself as either reinforcement or matrix for functional materials such as flexible transparent film,^27^ conductive polymers,^28^ and antibacterial textiles.^29^ It is also necessary to verify BC as building materials for flexible and cost‐effective NGs.

In this work, we fabricated a piezoelectric paper with piezoelectric BTO nanoparticles and BC through a facile vacuum filtration method. The BTO/BC piezoelectric paper based NGs showed an enhanced output performance compared to traditional BTO/PDMS based devices. The unique entangled BC nanofiber networks enable BTO nanoparticles to be dispersed in the piezoelectric paper uniformly, which makes the excellent piezoelectric property of the paper. The BTO/BC piezoelectric paper based NG can generate voltage of 1.5 V in bending conditions, which can drive a commercial liquid crystal display (LCD) screen. The flexible BTO/BC piezoelectric paper based NG is lightweight, eco‐friendly, and cost‐effective, which holds great promises for achieving wearable or implantable energy harvesters and self‐powered electronic devices.

## 2 Results and Discussion

**Figure 1:**
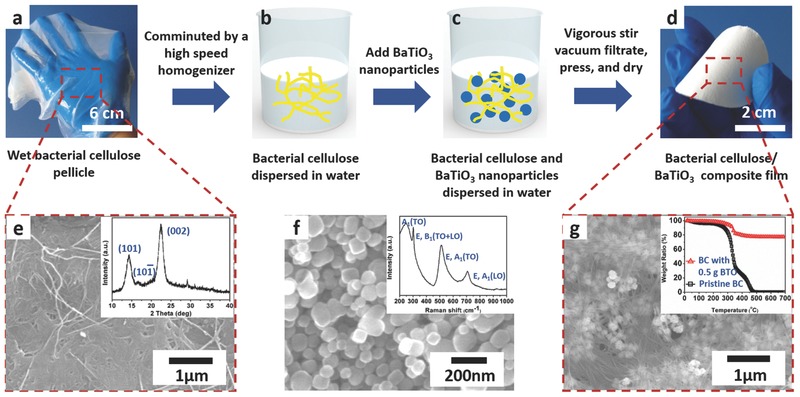
a–d) Fabrication process of the BTO/BC piezoelectric paper. e) An SEM image and X‐ray diffraction pattern (inset) of the pristine BC membrane. f) An SEM image and Raman spectrum (inset) of the BTO nanoparticles synthesized by hydrothermal method. g) An SEM image and thermal degradation behavior (inset) of the piezoelectric paper.

**Figure** **1**a–d shows the fabrication process of the BTO/BC piezoelectric paper. As can be seen in Figure 1a,e, raw BC are transparent gel‐like pellicles which are composed of cellulose nanofibers with the diameter of ≈10–30 nm and all the nanofibers are found to aggregate densely due to the loss of water. The X‐ray diffraction (XRD) pattern of the BC membrane is shown in the inset of Figure 1e. The diffraction peaks of 14.2°, 16.5°, and 22.5° are assigned to diffraction planes of (101), (10‐1), and (002) for native cellulose I, respectively.^30^ An aqueous suspension of BC fibers can be obtained by mechanically breaking the interconnected nanofiber networks with a high speed homogenizer. BTO nanoparticles were synthesized by hydrothermal method.^18^ As illustrated in Figure 1f, the average diameter of BTO nanoparticles is about 100 nm. Raman spectrum (inset of Figure 1f) shows peaks positioned at 250 cm^−1^ [*A*_1_(TO)], 302 cm^−1^ [*E*, *B*_1_(TO+LO)], 511 cm^−1^ [*E*, *A*_1_(TO)], and 710 cm^−1^ [*E*, *A*_1_(LO)], which represent a high piezoelectric tetragonal phase of BTO.^31^ The as‐synthesized BTO nanoparticles were ultrasonically dispersed in distilled water and then mixed with BC aqueous suspension under vigorous stirring to guarantee sufficient blending. BTO/BC piezoelectric paper can be formed by vacuum filtrating the blended suspension with a microporous membrane followed by a pressing and drying process. Figure 1g demonstrates the field‐emission scanning electron microscopy (FE‐SEM) image of the BTO/BC piezoelectric paper. It is found that the disintegrated BC nanofibers get associated again due to the strong interaction of hydrogen bonds and BTO nanoparticles are uniformly bounded within the BC matrix. Thermogravimetry analyses (TGA) in the inset of Figure 1g exhibits that pristine BC undergoes decomposition in two steps with degradation temperatures at around 350 °C (weight loss of 70%) and 450 °C (weight loss of 99%). Compared with the pure BC which has almost no mass residue after calcination, the BC mixed with 0.5 g BTO nanoparticles presents a mass residue of nearly 80% even above 500 °C, which indicates that the mass fraction of BTO nanoparticles in the paper is very high. This loading percentage is much higher than that achieved by a layer‐by‐layer approach (48 wt%).^32^ This distinct feature makes the BTO/BC piezoelectric paper as light as possible because most of the weight is concentrated on the piezoelectric component, which is an important merit for integration applications.

**Figure 2:**
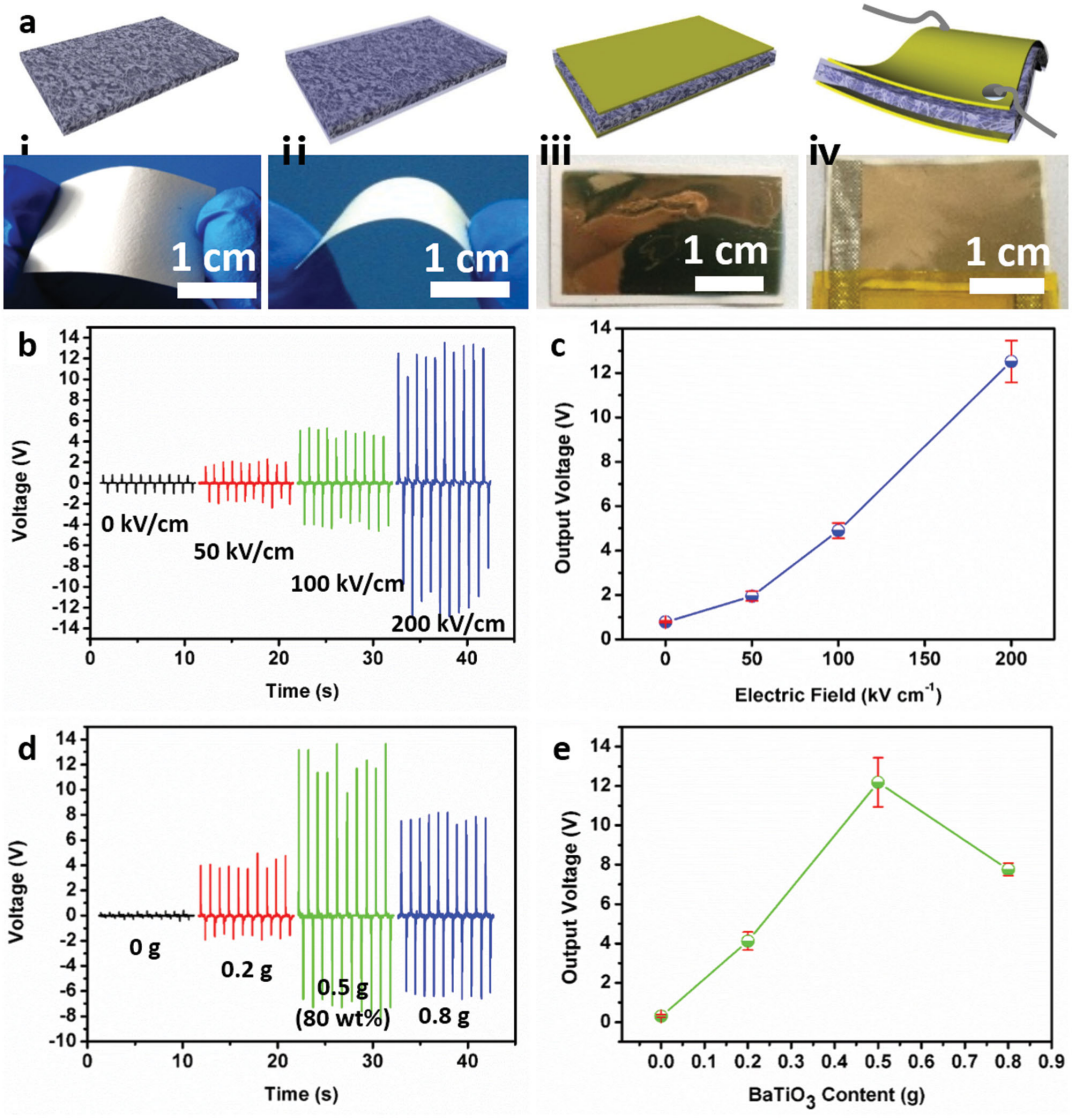
a) Fabrication process of the BTO/BC piezoelectric paper based NG device. b) The output voltage of the BTO/BC piezoelectric paper based NG with different poling electric field. c) Average output voltage dependence on poling electric field. d) The output voltage of the BTO/BC piezoelectric paper based NG with different BTO content. e) Average output voltage dependence on BTO content.

BTO/BC piezoelectric paper was used as an active layer to fabricate NGs, as shown in **Figure** **2**a. First, a thin layer of PDMS was spin‐coated on both side of the piezoelectric paper to provide a smooth surface for the electrodes and to protect the paper from moisture in the air. A layer of Ti/Au (10 nm/100 nm) was then deposited on both sides of the paper as electrodes. Two conductive tapes were then connected to the top and bottom electrodes, respectively. All the devices are characterized with the same active size of 3 × 2 cm^2^. Basically, poling process is of great importance for the BTO/BC piezoelectric paper because disordered dipoles in ferroelectric BTO domains need to be aligned by an external electric field so that the piezoelectric potential can be enhanced in a specific direction. To reveal the necessity of this process, a range of electric field were applied to pole the devices. As shown in Figure 2b,c, the unpoled device showed output voltage of only ≈1 V. As the poling electric field increased from 50 to 200 kV cm^−1^, the output voltage can be enhanced to more than 12 V. This can be ascribed to the rearrangement of the ferroelectric dipoles under high electric field. Furthermore, the output performance of the NG device can be affected by the amount of BTO nanoparticles contained in the BTO/BC piezoelectric paper. As shown in Figure 2d,e, when no BTO was contained in the paper, negligible output signals were detected which may originate from the capacitance change of the device. As the amount of BTO nanoparticles increasing from 0.2 to 0.5 g, the output voltage raised from ≈4 to ≈13 V. However, when BTO was further added to 0.8 g, the output voltage decreased to ≈8 V. This can be understood by the trade‐off between the density of piezoelectric points and the total permittivity of the piezoelectric paper. The increasing amount of BTO nanoparticles will undoubtedly provide more piezoelectric points in the paper which is helpful to generate high piezoelectric output. However, excessive amount of BTO nanoparticles can result in very high dielectric constant of the composite, which can weaken the electromechanical coupling effect of the piezoelectric paper.^33^

**Figure 3:**
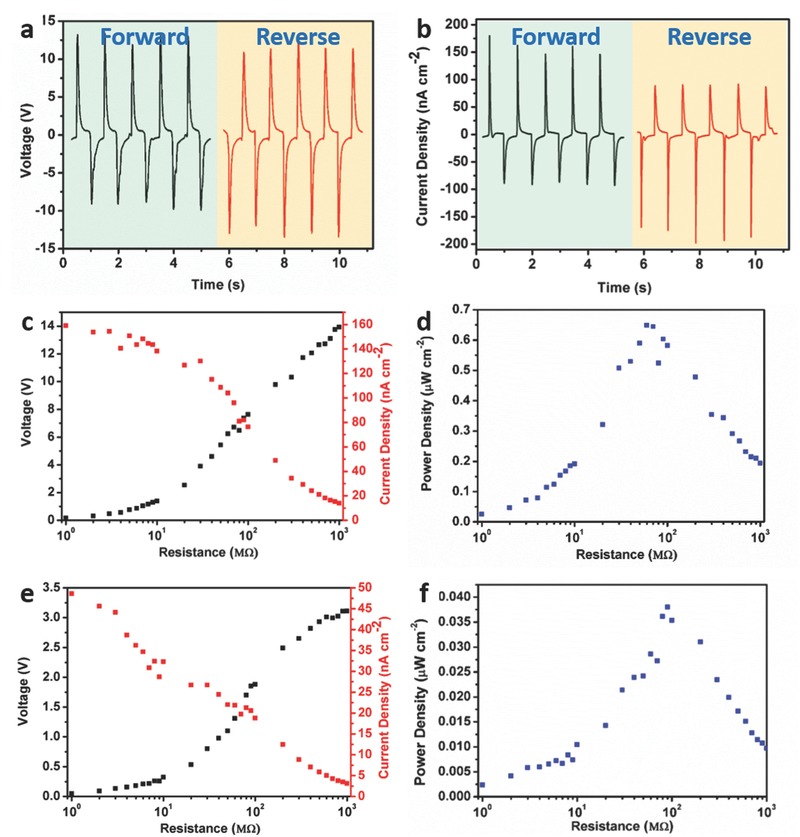
a) Open‐circuit voltage and b) short‐circuit current density of the BTO/BC piezoelectric paper based NG both in forward and reverse connections. The output voltage and c) current density and d) power density of the BTO/BC piezoelectric paper based NG with different external load resistance. The output voltage and e) current density and f) power density of the BTO/PDMS based NG with different external load resistance.

The output performance of the device is investigated by measuring the open‐circuit voltage and short‐circuit current when the NGs are subjected to cyclic compressive stress in the normal direction. As shown in **Figure** **3**a,b, the open‐circuit voltage and short‐circuit current density can reach as high as 14 V and 190 nA cm^−2^, respectively. The value discrepancy of each peak can be attributed to the different strain rate of the device during compressing and releasing process.^34^ To verify the signals are induced by the piezoelectric potential in response to the deformation of the piezoelectric paper, it is essential to conduct switching‐polarity test.^9^ In the reverse connection, the *V*–*t* signal exhibits a negative pulse followed by a positive pulse in response to a pressing and a releasing action with the average output voltage and current density maintained at similar magnitude with the forward connection. Therefore, the possible artifacts from triboelectricity and the measurement system can be ruled out. Figure 3c illustrates the dependence of the output characteristic on external load resistance. With the increment of the load resistance from 1 MΩ to 1 GΩ, the output voltage increases gradually from about 0.16 to 14 V, while the current density decreases from 160 to 14 nA cm^−2^. The output power density can be calculated by *V* ^2^/*R*, where *V* and *R* represent the output voltage and the corresponding external load resistance, respectively. As plotted in Figure 3d, the output power reaches the maximum value of 0.64 μW cm^−2^ at a matched resistance value of 60 MΩ.

**Figure 4:**
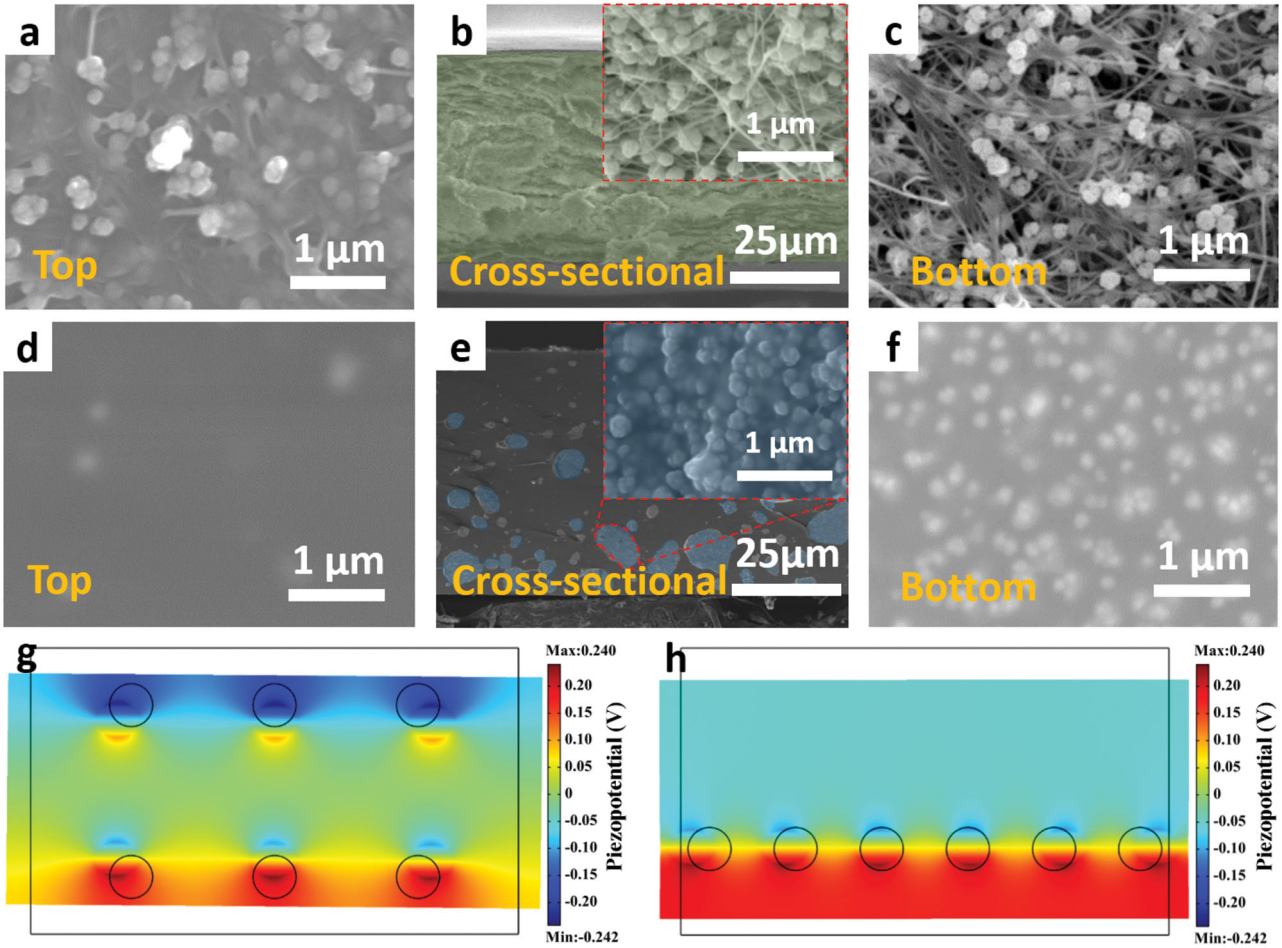
SEM image of the a) top surface, b) cross‐sectional, and c) bottom surface of the BTO/BC piezoelectric paper. SEM image of the d) top surface, e) cross‐sectional, and f) bottom surface of the BTO/PDMS film. COMSOL simulation results of the output voltage of the g) BTO/BC piezoelectric paper and h) BTO/PDMS film with the compressive stress of 0.1 MPa.

To understand the enhancement effect of this BTO/BC structure for the performance of NGs, we have also fabricated devices with commonly used BTO/PDMS structure. As exhibited in Figure 3e, both of the output voltage and current density are much lower than the BTO/BC based devices. The maximum output power generated by BTO/PDMS based devices is only 0.04 μW cm^−2^, which is more than ten times lower. To reveal the remarkable merit of using BC as matrix for BTO nanoparticles to disperse, SEM characterization is used to compare the top, bottom, and cross‐sectional structure of both kinds of the film. As shown in **Figure** **4**a–c, BTO nanoparticles are uniformly embedded in the whole BC matrix without any obvious aggregation. However, for BTO/PDMS film, limited amount of BTO nanoparticles are existed near the top surface of the film while extensive nanoparticles are accumulated at the bottom of the film. From the side view of the PDMS based film in Figure 4e, it is clear that most of the nanoparticles have aggregated into large clusters and distribute at the bottom of the PDMS body. Uniform dispersion of piezoelectric nanoparticles within a polymeric matrix is one of the key issues for piezoelectric film to yield high output. The piezopotential distributions inside the piezoelectric films are simulated by COMSOL software. As calculated by simplifying the film and BTO nanoparticles as a rectangular model and six piezoelectric circles in Figure 4g,h, it is predicted that homogeneous dispersion of BTO nanoparticles can lead to higher piezoelectric potential than the case that all the particles are distributed at the bottom of the matrix with other conditions unchanged. However, it is intrinsically difficult to disperse BTO nanoparticles into sticky PDMS for inevitable aggregation and settlement will happen before the whole body has fully cured. The percolated network of the BC nanofibers enables well dispersion of piezoelectric nanoparticles in the film; thus, the piezoelectric output can be optimized. Furthermore, cellulose is reported to have a Young's modulus in the range 78 ± 17 GPa,^35^ which is properly higher than PDMS (1–2 MPa).^18^ The stiff nanofibers can transfer stress to the localized piezoelectric BTO nanoparticles effectively. As a result, the BTO nanoparticles will be deformed more significantly in the BC matrix and enhanced output signals can be yielded.

**Figure 5:**
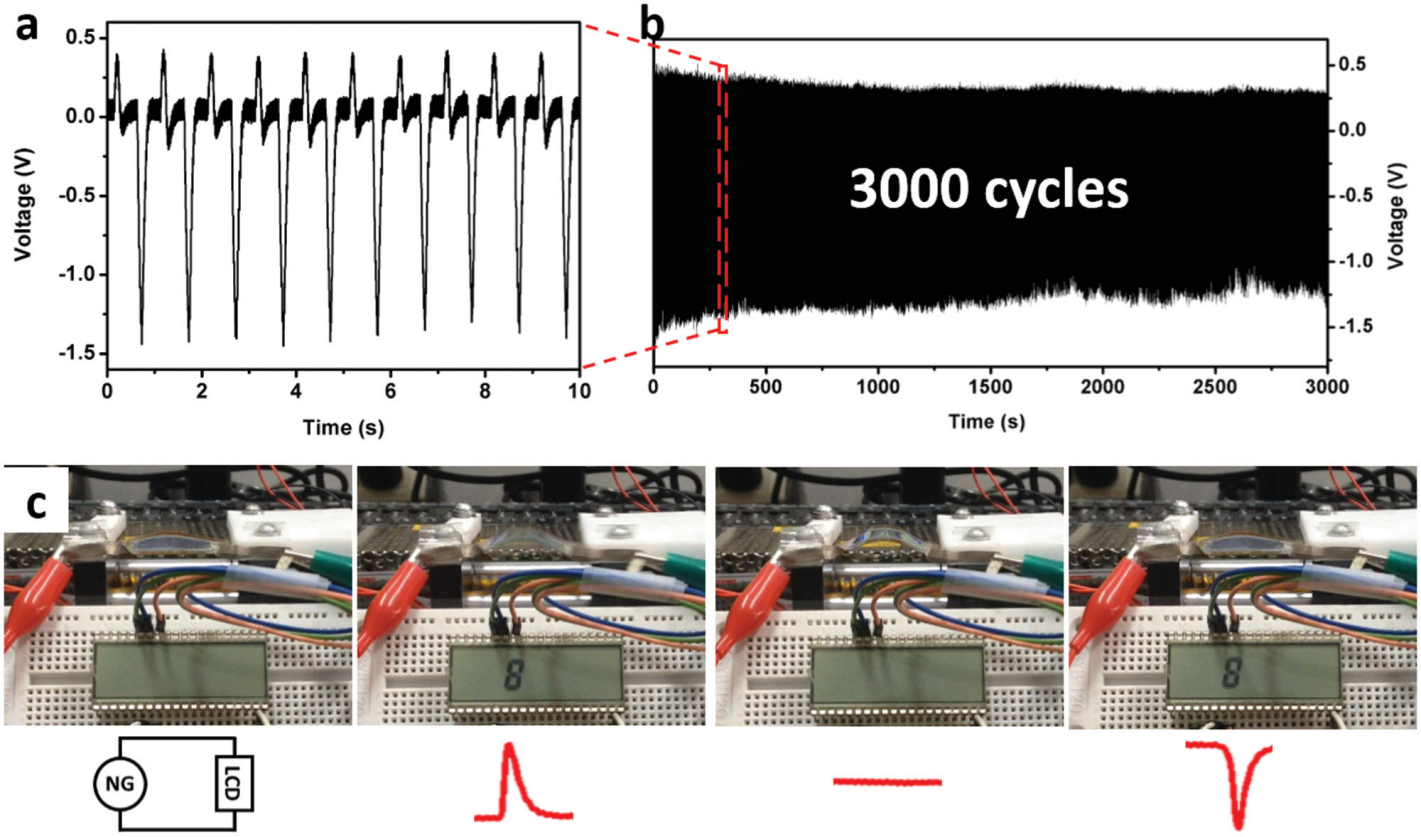
a) Output voltage of the BTO/BC piezoelectric paper based NG under the bending frequency of 1 Hz. b) Cyclic bending test for 3000 cycles of bending/releasing motions. c) Four states when a commercial LCD screen is driven by the NG under bending conditions.

To demonstrate the potential applications requiring flexible properties of the NG device, the output performance was measured in bending/releasing conditions. The NG device was mounted on a Kapton film which was bent and released repeatedly by a bending stage. As shown in **Figure** **5**a, a peak output voltage of 1.5 V was achieved with the deformation frequency of 1 Hz. Furthermore, the durability of the device was also examined by applying bending/releasing repeatedly (Figure 5b). It was found that at the beginning of the test, minor degradation of output voltage was observed, which was caused by small relative displacement between fixture and the device. The output voltage was maintained at about 1.2 V afterward and no obvious performance decline can be seen for up to 3000 cycles, indicating excellent robustness of the device. The BTO/BC piezoelectric paper based NGs can be applied to harvest mechanical energy and operate small electronics. A commercial LCD can be directly triggered by cyclic bending/releasing the device. Since the LCD is a nonpolar device, it can be driven by AC source without a rectifier unit. Both the bending and releasing action of the finger can generate the flash of number “8” on the screen, while no number is displayed during the holding process (shown in a video, Supporting Information).

## 3 Conclusion

In this work, we demonstrate a piezoelectric paper by incorporating BaTiO_3_ nanoparticles into bacterial cellulose nanofiber networks to realize lightweight, flexible, and cost‐effective NGs. The piezoelectric paper is fabricated by a vacuum filtrating method, which is facile and scalable. The BTO/BC piezoelectric paper based NG shows outstanding output performance with the open‐circuit voltage of 14 V and short‐circuit current density of 190 nA cm^−2^. The maximum power density generated by this unique BTO/BC structure is 0.64 μW cm^−2^, which is more than ten times higher than BTO/PDMS structure. This enhancement can be ascribed to homogeneous distribution of piezoelectric BTO nanoparticles in the BC matrix, which is implemented by the percolated networks of BC nanofibers. The BTO/BC piezoelectric paper based NGs also represent potential applications as flexible energy harvesters. In the cyclic bending condition, the device can generate a peak voltage of 1.5 V with high stability and durability. A commercial LCD screen can be driven by the cyclic generated power. The flexible BTO/BC piezoelectric paper based NG is lightweight, eco‐friendly, and cost‐effective, which holds great promises for achieving wearable or implantable energy harvesters and self‐powered electronic devices.

## 4 Experimental Section

*Preparation of the BTO/BC Piezoelectric Paper*: A high speed homogenizer was used to mechanically break the interconnected BC nanofiber networks with the speed of 10 000 rpm for 30 min. An aqueous suspension with BC fibers uniformly dispersed can be obtained. Hydrothermal BaTiO_3_ nanoparticles were ultrasonically dispersed in distilled water and then mixed with BC aqueous suspension. The well‐mixed dispersions were vacuum filtrated by a microporous membrane (Jingteng, 0.22 μm, Tianjin, China). At last, the filtrated film was pressed by a 2 kgf load and dried at 70 °C for 24 h to get the piezoelectric paper.

*Characterization and Measurements*: An FE‐SEM (Quanta3DFEG), X‐ray diffractometer (TTRIII), and Raman spectroscopy (JY‐HR800) with an Ar^+^ laser source were used for materials characterization. Cyclic bending deformation was provided by a home‐made bending stage. The open‐circuit voltage signal was recorded by a digital oscilloscope (DS4052, RIGOL) and a low‐noise voltage preamplifier (Standard Research System Model SR560). The short‐circuit current signal was recorded by a low‐noise current preamplifier (Standard Research System Model SR570).

## Supporting information

As a service to our authors and readers, this journal provides supporting information supplied by the authors. Such materials are peer reviewed and may be re‐organized for online delivery, but are not copy‐edited or typeset. Technical support issues arising from supporting information (other than missing files) should be addressed to the authors.

Supplementary
